# Exostose géante de l'extrémité inférieure du femur

**DOI:** 10.11604/pamj.2014.18.233.4864

**Published:** 2014-07-22

**Authors:** Younes Ouchrif, Issam Elouakili

**Affiliations:** 1 Service de Chirurgie Orthopédique, CHU de Rabat, Maroc

**Keywords:** Exostose, formations osseuses, cartilage, genou, Exostosis, bone formation, cartilage, knee

## Image en medicine

Les exostoses sont des formations osseuses développées à la surface des os et qui sont recouvertes par du cartilage. Il s'agit de la plus fréquente des tumeurs bénignes après les fibromes non ossifiant et les lacunes métaphysaires. Elles se localisent préférentiellement sur le versant métaphysaire du cartilage de croissance fertile. Sur le plan physiopathologique l'hypothèse d'une bascule à 90° d'un fragment du cartilage de croissance est appuyée par les travaux expérimentaux d'Ambrosia et Ferguson. La découverte fortuite est une circonstance diagnostique fréquente quand l'exostose est de petite taille, elle est alors constatée lors d'un bilan radiologique réalisé pour un autre motif. Les formes symptomatiques sont représentées par les formes douloureuses, celle-ci peut avoir plusieurs explications: fracture de la base de l'exostose, bursite, distension des parties molles. Les formes volumineuses, c'est le cas de notre patient, doivent suspecter chez l'adulte une dégénérescence. Le diagnostic de certitude est fait par la radiographie standard qui montre une image d'addition sessile ou pédiculée, précise son siège, son volume et la présence de micro calcifications. La TDM a peu d'intérêt dans l'exploration de l'exostose solitaire. L'IRM a pour intérêt d’étudier les rapports de la tumeur avec les éléments nobles vasculo nerveux dans le cadre d'un bilan pré opératoire. La confirmation diagnostique est anatomopathologique. Le risque de dégénérescence sarcomateuse est évalué dans la littérature à 1% et se fait le plus souvent vers un chondrosarcome, doit être suspecté quand l'exostose augmente rapidement de volume. Le traitement est chirurgical et consiste en une résection complète de l'exostose à partir de sa base, la biopsie préalable n'est pas systématique dans la forme typique.

**Figure 1:**
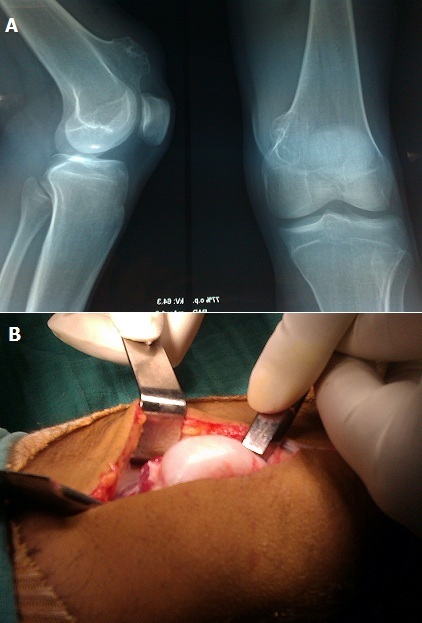
A) radiographie du genou droit de face et de profil montrant une exostose de l′extrémité inférieure du fémur sessile; B) image per opératoire montrant l′exostose géante

